# Enhancement of Hydration Activity and Microstructure Analysis of γ-C_2_S

**DOI:** 10.3390/ma16206762

**Published:** 2023-10-19

**Authors:** Ziyue Yan, Yaqing Jiang, Kangting Yin, Limeng Wang, Tinghong Pan

**Affiliations:** 1 College of Mechanics and Materials, Hohai University, Nanjing 210098, China; yanziyue@hhu.edu.cn (Z.Y.); ktying@hhu.edu.cn (K.Y.); 211308020023@hhu.edu.cn (L.W.); 2 Key Laboratory of Disaster Reduction in Civil Engineering, Faculty of Civil Engineering and Mechanics, Kunming University of Science and Technology, Kunming 650500, China

**Keywords:** γ-C_2_S, nano-SiO_2_, hydration, C/S ratio

## Abstract

This paper investigated the combined effect of chemical activators and nano-SiO_2_ on the hydration reaction and the microstructure of γ-C_2_S. The hydration reaction of γ-C_2_S slurry activated with chemical activators (NaHCO_3_, NaOH, K_2_CO_3_, and KOH at 1 mol/L) was enhanced by 1% nano-SiO_2_. The hydrate reaction rate was determined by isothermal calorimetry, and the hydrated samples were characterized by XRD, TGA/DTG, SEM-EDS, and ^29^Si MAS/NMR. The results revealed a substantial enhancement in the hydration activity of γ-C_2_S due to the presence of the alkaline activator. Furthermore, nano-SiO_2_ did not alter the composition of γ-C_2_S hydration products, instead providing nucleation sites for the growth of hydration products. Incorporating nano-SiO_2_ promoted the formation of C-(R)-S-H gel with a low calcium-to-silica ratio and increased its polymerization levels, resulting in more favorable structures. Among all the activators used in this study, potassium salts had a better activation effect than sodium salts. After 28 days of curing, the degree of hydration reaction in the KC+Si group was 48% and about 37% for the NHC+Si group. Whereas, the KH+Si and NH+Si groups only reached approximately 20% after the same hydration duration.

## 1. Introduction

Carbon dioxide emissions significantly contribute to the greenhouse effect, sea level rise, and climate anomalies. Moreover, cement clinker preparation is a significant source of GHG (greenhouse gas) emissions [1]. Cement production emits at least 2.1 billion tons of carbon dioxide (CO_2_) annually, contributing approximately 7% to the total global emissions [2]. Therefore, an immediate requirement exists for low-energy and low-emission production methods to mitigate the environmental issues stemming from cement preparation. High-belite cement, with dicalcium silicate (Ca_2_SiO_4_) as its primary mineral, is renowned for its low energy consumption and low carbon emissions. This cement variant features a lower calcination temperature and duration, effectively reducing carbon emissions during production [3].

Dicalcium silicate (C_2_S) contains five well-established polymorphs: γ, β, αL, αH, and α′, which have various hydration activities [4]. The water-reactive β-C_2_S is the main mineral component of cement, while the γ-C_2_S is non-hydration active [5]. β-C_2_S undergoes transformation to γ-C_2_S at temperatures lower than 500 °C. This process involves heightened internal stresses with a volume expansion of approximately 12%, causing fragmentation into fine particles, a phenomenon known as “dusting” [6]. To prevent the transition of β-C_2_S to γ-C_2_S, the preparation process typically follows two routes: one involves the addition of suitable doping ions (e.g., aluminum) before firing; the other entails rapid cooling of the clinker after the firing process is finished, which increases the complexity of the preparation process. Due to its uncomplicated preparation process, self-powdering properties, and low carbon emissions, γ-C_2_S is a promising low-carbon building material [1]. As a result, enhancing the hydration activity of γ-C_2_S is a strategic objective in developing low-carbon cement.

Up to now, numerous scholars have investigated the impact of various chemical additives on the hydration properties of C_2_S with different crystal types. It has been observed that incorporating alkaline salts can accelerate the hydration rate of C_2_S, thereby enhancing the formation of C-S-H gels. Kriskova et al. [6] investigated the effects of NaOH and Na_2_CO_3_ on the hydration properties of γ-C_2_S. They indicated that elevated concentrations of activators (with Na_2_O content of 4% and 8%) enhanced the hydration activity of γ-C_2_S, resulting in increased mechanical strength for both hydrated pastes at 90 days. Warda Ashraf [7] further investigated the microstructures of γ-C_2_S pastes in the presence of a high concentration of sodium salt (10% Na_2_O content). They demonstrated that NaHCO_3_ activated the hydration activity of γ-C_2_S more effectively than other sodium salts. Zhang [8] and Nisrine [9] explored the accelerating effect of K_2_CO_3_ and KOH on C_2_S hydration. The retention mechanisms of potassium and the nature of the hydration product potassium C-(K)-S-H were clarified. Nonetheless, their research did not explore the impact of potassium salt activators on the hydration properties of γ-C_2_S. These studies demonstrated that K^+^ and Na^+^ not only activate the hydration activity of C_2_S but also substitute Ca^2+^ in C-S-H gels, promoting the precipitation of C-(R)-S-H.

Although chemical activators can effectively enhance the hydration activity of γ-C_2_S, most current studies utilize high concentrations of activators, which pose the risk of environmental contamination. γ-C_2_S is also challenging to activate with low concentrations of activators. On the other hand, nanomaterials have been extensively used in cementitious materials, capturing the attention of many scholars [10,11,12]. Due to the seeding effect and pozzolanic effect of nanomaterials, they can enhance the pore structures and increase the compactness of cementitious materials, thereby effectively improving the development of strength [13,14]. L.P. Singh et al. [15] explored the initial hydration property of highly hydrated active tricalcium silicate (C_3_S) in the presence of nano-SiO_2_. The hydration induction period of tricalcium silicate was accelerated, and the C-S-H gels with a low calcium-silicon (C/S) ratio were formed in the presence of nano-silica. Yang et al. [16] investigated the effect of titanium dioxide (TiO_2_) nanoparticles on enhancing the hydration characteristics of single minerals in cement. The research showed that although the incorporation of nano-TiO_2_ accelerated the hydration reaction of C_2_S, the resulting enhancement effect was not significant due to the low initial hydration reaction of C_2_S. Thomas et al. [17] investigated the influence of water glass (NaSi) and C-S-H seeds on the reaction kinetics of C_2_S. The study revealed that incorporating C-S-H seeds within the NaSi activation system could enhance the hydration process of C_2_S, thereby validating the potential for a synergistic interaction between the chemical activator and the crystal seed additive. AlTawaiha et al. [18] conducted a comprehensive investigation into the influence of nano-silica (NS) on the mechanical properties of concrete. They demonstrated that NS could decrease micropores within the structure, thereby enhancing the mechanical properties of concrete. Golewski [19] reported the combined effect of coal fly ash and nano-SiO_2_ on concrete composites. Nano-SiO_2_ filled the pores in the cement structure through the filler effect. Due to its high specific surface and high activity, the cementitious material undergoes a secondary hydration reaction to form a denser structure. In these experiments, the combined use of coal fly ash and nano-SiO_2_ had synergistic and positive effects on improving the microstructure of eco-friendly concretes. However, the seeding influence of nanomaterials is exerted exclusively on cementitious materials with hydration activity, while γ-C_2_S lacks hydration activity. Therefore, it can be predicted that nanomaterials with a single doping cannot promote the hydration of γ-C_2_S. In addition, a few existing reports exist on the synergistic effect of chemical activators and crystal species on γ-C_2_S. Based on the above discussions, this paper investigates the synergistic impacts of the seeding effect and chemical activation on the hydration properties of γ-C_2_S and the changes in its microstructure.

In this study, a low-alkalinity chemical activator was employed to activate the hydration activity of γ-C_2_S, along with introducing nano-silica to promote hydration. The main objective was to investigate the viability of activating environmentally friendly low-carbon cementitious materials, primarily belite clinker minerals. The specific goals of this paper include the following: (1) to comprehend the impact of different alkaline activators (i.e., NaHCO_3_, NaOH, K_2_CO_3_, and KOH) on the hydration reaction of γ-C_2_S, (2) to uncover the effect on the microstructures of activated pastes in the presence of nano-silica, and (3) to delineate the effect of Na^+^ or K^+^ and nano-SiO_2_ on the morphology, structure, and composition of C-S-H gels.

## 2. Materials and Methods

### 2.1. Synthesis of γ-C_2_S and Characterization

**Figure 1:**
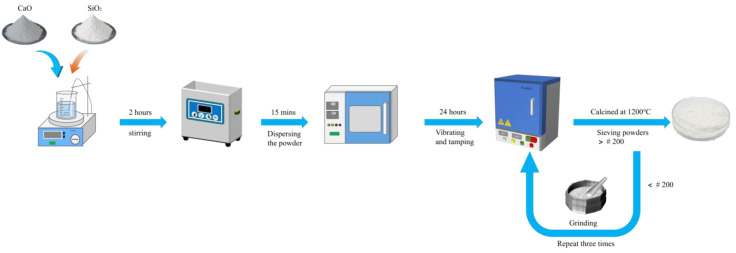
Flow chart for the synthesis of γ-C_2_S.

The γ-C_2_S was synthesized using the solid-phase reaction method with AR (analytical reagent) grade CaO and SiO_2_ provided by Aladdin^®^ as raw materials. The preparation process involved several steps: initially, the CaO and SiO_2_ were weighed at a molar ratio of 2:1. Next, they were mixed with an alcohol solution, subjected to 15 min of ultrasonic mixing, and stirred for 2 h using a magnetic stirrer to ensure uniformity. The mixture underwent drying at 60 °C in an oven, followed by thorough grinding using a mortar and pestle, resulting in the acquisition of a uniformly blended raw material. Then, the prepared raw material was compacted, placed into a muffle furnace, and heated to 1200 °C at a rate of 5 °C/min. It was then held at this temperature for 5 h and left on the stove until it reached room temperature (to ensure that β-C_2_S was fully converted to γ-C_2_S). Ultimately, the resultant material was sieved using a # 200 (74 μm) sieve and refired (three times in total) to ensure a complete reaction between CaO and SiO_2_. Figure 1 shows the flow chart for the synthesis of γ-C_2_S.

**Figure 2:**
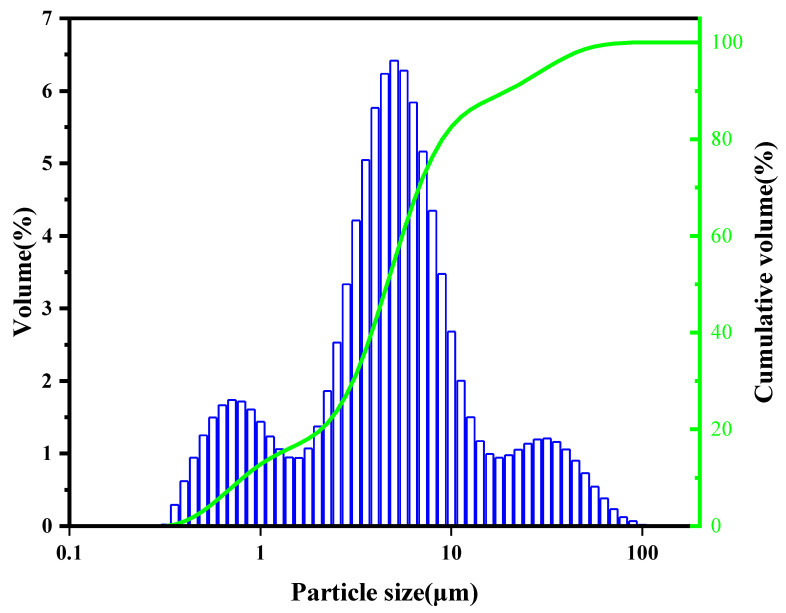
Particle size distribution of the synthesized γ-C_2_S powder.

**Figure 3:**
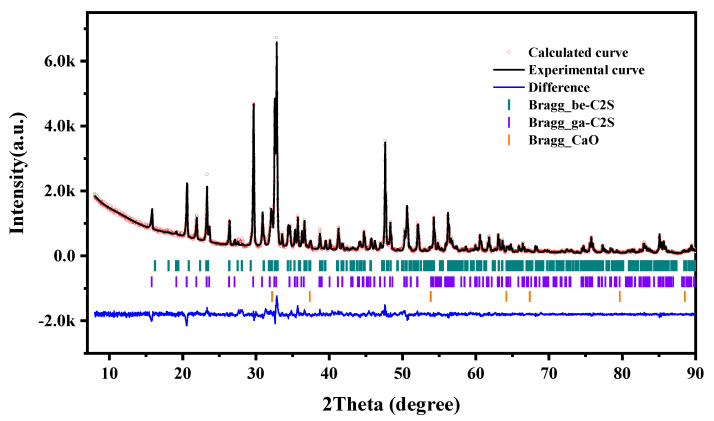
The XRD pattern of synthetic γ-C_2_S refined by Rietveld refinement.

The particle size distribution of the synthesized γ-C_2_S powder was determined using a Malvern Mastersizer particle size analyzer. The results are illustrated in Figure 2. The d_50_ and d_90_ of γ-C_2_S powder were 5 μm and 20 μm, respectively. The clinker underwent quantitative analysis using the XRD Rietveld method to confirm the purity of the synthesized γ-C_2_S (given in Figure 3). Based on the XRD data, the crystal structure of the sample was quantitatively analyzed by the Rietveld method, revealing that the mixture consists of 89% γ-C_2_S and 11% β-C_2_S. The negligible presence of calcium oxide and silica in the powder proves the completeness of the solid-phase reaction.

Four different chemical activators, including NaOH, NaHCO_3_, KOH, and K_2_CO_3_ (addressed as NH, NHC, KH, and KC, respectively, for the remainder of the paper), were employed in this study. The alkaline salts were dissolved in distilled water to achieve a 1 mol/L concentration. SiO_2_ nanoparticles (20 nm) served as the seed activator. The liquid-to-binder (l/b) ratio was 1.0, and the amount of seed activator was 1% of the total mass of γ-C_2_S. The nano-SiO_2_ was first mixed with γ-C_2_S before adding a prepared chemical solution. The test samples were hydrated in sealed bottles for 7 and 28 days. They were then removed after the specified ages. The hydration reaction was terminated using an anhydrous ethanol solution, followed by drying in a vacuum oven at 60 °C for at least 2 days. Ultimately, the samples were examined by X-ray diffraction (XRD), thermogravimetric analysis (TGA/DTG), scanning electron microscope (SEM), and nuclear magnetic resonance (^29^Si MAS/NMR).

### 2.2. Testing Methods

Isothermal calorimetry was used to study the early hydration exotherm of samples at 20 °C (TAM AIR, TA INSTRUMENTS, 159 Lukens Dr, New Castle, DE, USA, DE 19720). First, 5 g of powder sample and 0.05 g of nano-SiO_2_ were weighed and mixed thoroughly. Next, the sample was mixed with the solution at a mass ratio of 1 in an isothermal calorimeter bottle, stirred with a needle for about 30 s, and then placed in the isothermal calorimeter. The exothermic data of the hydrated samples were collected at 20 °C for 3 days.

XRD (RIGAKU SMARTLAB 3 KW, 196-0003 3-9-12 Matsubara-cho, Akishima-shi, Tokyo, Japan) was used to characterize the hydration products of the samples at different ages, with a scanning speed of 5 °C/min and a scanning range of 5–90° 2θ.

Thermogravimetric analysis (TGA) of samples was performed using a thermogravimetric analyzer (TA Q600, Newcastle, DE, USA). After reaching the specified age, the samples stopped the hydration reaction and then were ground to pass through a # 200 sieve, and then the powdered samples were raised from room temperature to 1000 °C at a rate of 10 °C/min in a constant nitrogen (N_2_) atmosphere.

The microscopic morphologies of hydration products were observed using a scanning electron microscope (TESCAN MIRA LMS, KOHOUTOVICE, Brno, Czech Republic) at an accelerating voltage of 20 KV and a working distance of 11 mm. Prior to imaging, the specimen blocks, which had been cured for 28 days, were stored in anhydrous ethanol for 7 days to stop further hydration reactions. After solvent-vacuum drying for 1 day, the samples were adhered to the sample stage using conductive adhesive and sprayed with gold on their surface before being put under the SEM. The energy-dispersive spectrum (EDS) was used to determine the information related to the atomic fractions of individual elements (i.e., C, Ca, Si, and O).

^29^Si MAS NMR curves were obtained using a JEOL JNM-ECZ600R NMR spectrometer in Osaka-fu, Tokyo, Japan. ^29^Si MAS NMR spectra with a resonance were obtained at a frequency of 99.29 MHz and a spinning speed of 15 KHz.

## 3. Results and Discussion

### 3.1. Heat of Hydration Analysis

**Figure 4:**
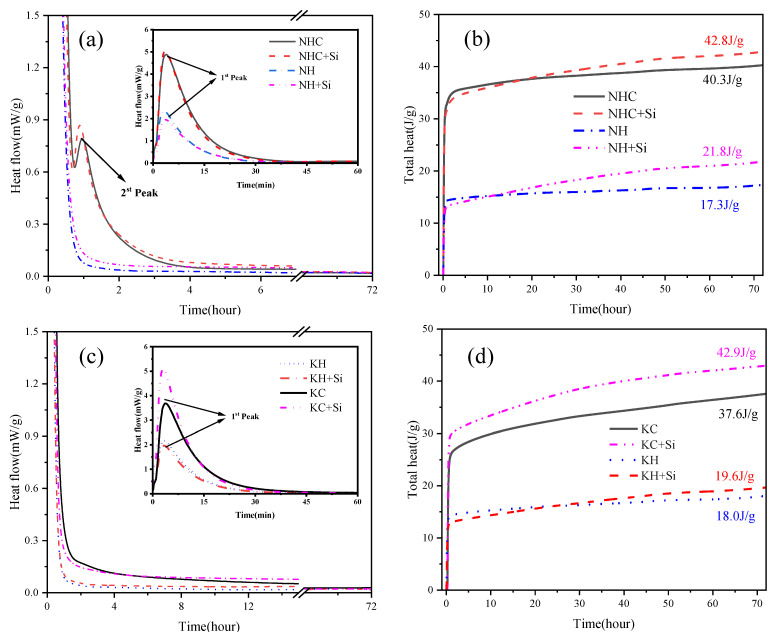
(**a**,**b**) Normalized heat flow and normalized heat of the γ-C_2_S hydration in Na^+^ activation system, (**c**,**d**) Normalized heat flow and normalized heat of the γ-C_2_S hydration in K^+^ activation system.

Figure 4 displays the heat flow and cumulative heat release curves of γ-C_2_S powder in various activation systems, along with the mass normalization of γ-C_2_S powder in activation systems.

As shown in Figure 4a, all activation systems exhibit a distinct initial exothermic peak during early hydration, corresponding to the exothermic behavior of the dissolution and the immediate reaction of the dry powder [20]. The broader and higher intensity of the first exothermic peak in the NHC activation system indicates that the NHC activator significantly enhances the dissolution rate of γ-C_2_S. The addition of nano-SiO_2_ amplifies the intensity of the first exothermic peak, agreeing well with findings by Althoey [21]. A secondary exothermic peak is also observed in the NHC and NHC+Si-activated samples, which is attributed to the generation of the hydration product C-(R)-S-H gel [20] (R denotes an alkali metal ion bound into the C-S-H structure (Na and K)). However, this peak is absent in the less-activated NH activation system. This discrepancy implies the effective enhancement of γ-C_2_S hydration activity by NHC compared with NH. Figure 4b reveals that after 3 days of hydration, the highest total heat release is found in the NHC+Si-activated paste (42.8 J/g), followed by NHC (40.3 J/g) and NH+Si-activated paste (21.8 J/g). In contrast, the NH-activated samples display the least accumulative heat release (17.3 J/g). The disparity in total heat release suggests that NHC activators exhibit greater efficacy than NH activators. This conclusion is in agreement with Warda Ashraf et al. [7]. In the early stage of the reaction, the exothermic heat of the nano-SiO_2_ activation system is typically lower than that of the control group. This phenomenon can be due to the huge specific surface area of nano-SiO_2_, resulting in higher kinetics. The nano-SiO_2_ can interact with Ca^2+^ released from γ-C_2_S grains during early hydration, which results in the generation of secondary C-(R)-S-H, thereby diminishing the solubility of Ca^2+^ and attenuating its hydration properties [15].

Figure 4c,d display the variation in hydration heat between KC- and KH-activated pastes with and without adding nano-SiO_2_. A prominent and broad first exothermic peak is observed in Figure 4c [7]. The first exothermic peak of the KC+Si-activated paste is significantly higher than that of other activation groups after mixing the nano-SiO_2_. During the induction period, the hydration heat release rate of the KC+Si-activated paste is lower than that of the KC-activated paste in the first 3–5 h of hydration and subsequently exceeds after 5 h. However, it maintains a noticeable heat release rate (0.15 mW/g) throughout the induction period. This indicates that the consumption of Ca^2+^ is accelerated more effectively by KC, leading to the fast precipitation of hydration products like C-(R)-S-H gel and CaCO_3_ [8]. In the KH activation system, the exothermic rate of the hydration process and the cumulative hydration exothermic amount are still unsatisfactory, indicating that OH^−^ encounters more significant challenges in activating the non-hydrated γ-C_2_S system.

Similar to alkali activation, chemical activation accelerates the dissolution rate of reactants, the precipitation of products, and the generation of hydration products by modifying the pH value of the hydrated slurry. As for NH and KH, the higher pH leads to a decrease in the solubility of Ca^2+^ in the hydrated slurry, consequently restricting the hydration rate. Nevertheless, NHC and KC release CO_3_^2−^, which increases the pH and accelerates the dissolution of Ca^2+^ (reacting with CO_3_^2−^ to form CaCO_3_), thereby promoting hydration [7]. Moreover, the total cumulative exothermic amount increases for each hydration sample in the presence of nano-SiO_2_, indicating the enhanced hydration degree of the activation groups by nano-SiO_2_.

### 3.2. XRD

**Figure 5:**
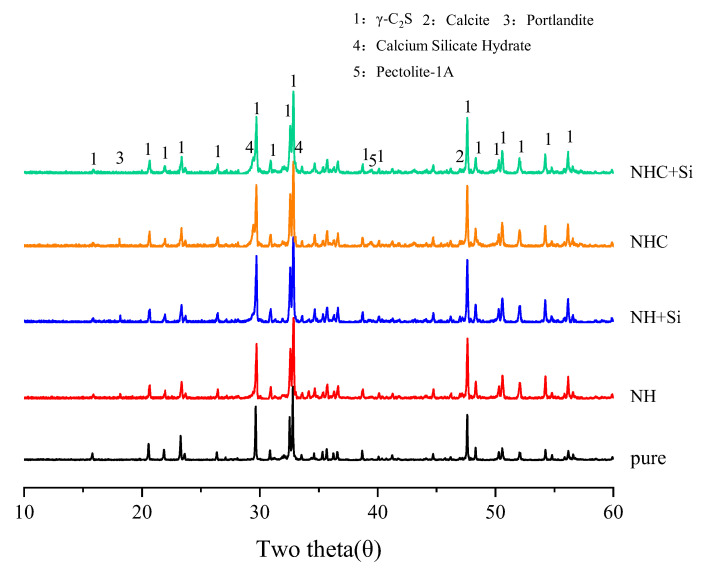
XRD diffractogram of hydrated γ-C_2_S with the Na^+^ activation system at 28 d.

**Figure 6:**
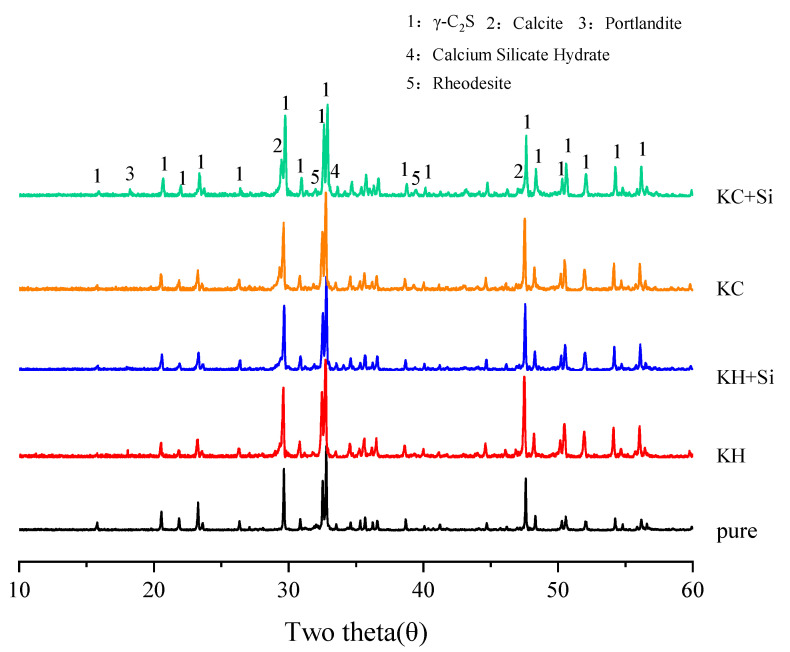
XRD diffractogram of hydrated γ-C_2_S with the K^+^ activation system at 28 d.

The XRD diffractogram of γ-C_2_S slurry activated with the Na^+^ activation system is shown in Figure 5, while the XRD diffractogram of γ-C_2_S slurry activated with the K^+^ activation system is presented in Figure 6. The XRD analysis indicates that the primary hydration products include portlandite, calcite, and C-(R)-S-H gel, and the addition of nano-SiO_2_ does not change the type of the products. During the hydration process, the intensity of the γ-C_2_S diffraction peaks in all samples is observed to decrease. A new peak appears around 29° 2θ, which can be attributed to either calcite or C-(R)-S-H (the presence of calcite and C-(R)-S-H on the same peak). As shown in Figure 5, after 28 days of hydration, NH, NH+Si, and NHC samples exhibit a novel and prominent diffraction peak at 18° 2θ, indicating the presence of portlandite (Ca(OH)_2_). However, in the NHC+Si-activated paste, the intensity of the Ca(OH)_2_ peak flattens out at 28 days, indicating the occurrence of a pozzolanic effect during this period. It is noteworthy that, in contrast to the findings of Warda Ashraf [7] and Kriskova et al. [6], gaylussite (Na_2_Ca(CO_3_)_2_·5H_2_O) is absent in the NHC activation system. The formation of gaylussite is caused by the carbonization of residual alkali in the pore solution. However, probably due to the low content of the alkali solution used in this study, the alkali is consumed during the hydration process, resulting in gaylussite not appearing as expected.

As illustrated in Figure 6, the KC-activated paste shows a more pronounced decrease in the prominent γ-C_2_S peak, accompanied by an elevated peak at 29° 2θ, indicating that γ-C_2_S can be activated by the KC activator more effectively. Furthermore, the intensity diffraction peak observed around 39.5° 2θ is identified as a new phase of potassium-containing Rhodesite KCa_2_(Si_8_O_18_(OH))6H_2_O (JCPDS card No. 01-076-2053) and Pectolite-1A HNaCa_2_Si_3_O_9_ (JCPDS card No. 01-076-0951), respectively, demonstrating the involvement of K^+^ and Na^+^ in the hydration of γ-C_2_S [8,22].

### 3.3. Thermal Analysis

**Figure 7:**
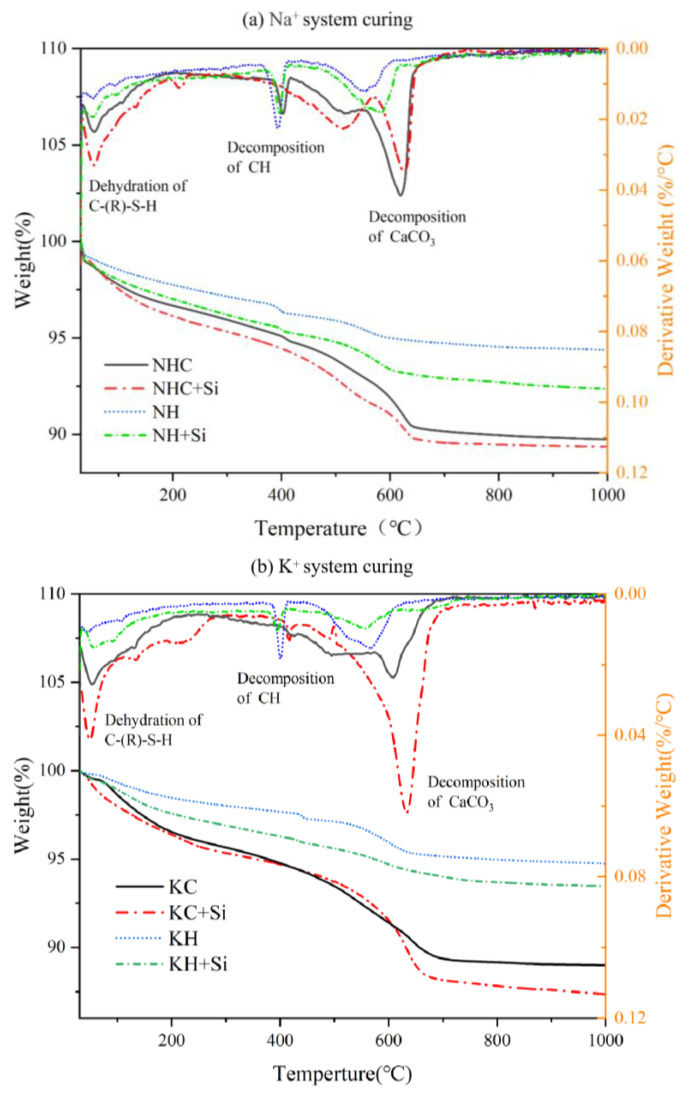
TGA/DTG results of chemically activated γ-C_2_S pastes. (**a**) The curve of Na^+^ activation system. (**b**) The curve of K^+^ activation system.

The TG-DTG curves of 28-day hydrated samples are exhibited in Figure 7a,b. Whether nano-SiO_2_ is present or absent, the samples experience an initial weight loss at around 100 °C, which can be attributed to the dehydration of the hydration product C-(R)-S-H gel [23]. Calcium hydroxide typically decomposes at approximately 400 °C [24]. The last weight loss peak (500–700 °C) can be attributed to the volatilization of CO_2_ from the decomposition of CaCO_3_. In the NHC and KC activation systems, the formation of the hydration product CaCO_3_ is primarily attributed to carbonate ions in the activator. In contrast, in the NH and KH activation systems, CaCO_3_ is mainly formed through the carbonation of Ca(OH)_2_ [23]. Multiple DTG peaks in the range of 500 °C to 700 °C for different γ-C_2_S activation groups are attributed to the formation of multiple crystalline forms of CaCO_3_ [25].

As shown in Figure 7a, a new mass loss is observed for NHC+Si-activated paste in the range of 200–300 °C, which can be caused by the decomposition of sodium silicate and amorphous calcium carbonate (calcium carbonate formed by the carbonation of C-(R)-S-H) [22]. The increase in mass loss before 100 °C in NHC+Si-activated paste and the absence of mass loss at 400 °C are attributed to the pozzolanic effect of nano-SiO_2_, which consumes Ca(OH)_2_ and promotes the generation of C-(R)-S-H [26]. A new mass loss is observed in the NHC+Si-activated paste at around 500 °C, indicating the decomposition of amorphous calcium carbonate [22]. As shown in Figure 7b, an additional peak is observed in the K^+^ activation system below 200 °C, which is attributed to the decomposition of amorphous calcium carbonate. Furthermore, the figure demonstrates that CaCO_3_ formed by the carbonization of Ca(OH)_2_ tends to decompose around 550 °C, whereas CaCO_3_ formed during hydration decomposes at a higher temperature of about 650 °C.

Compared to the control group, the exothermic peak of CH decreases in the SiO_2_ group, while the C-(R)-S-H peak increases. This observation implies that the pozzolanic effect of SiO_2_ nanoparticles consumes more Ca(OH)_2_, which facilitates its conversion into the hydration product C-S-H. The addition of nano-silica sharpens the exothermic peak at 600 °C, which indicates that more CaCO_3_ is generated. This phenomenon suggests that nucleation occurs at this point [27]. Furthermore, the higher degree of hydration of pastes containing nano-SiO_2_ (greater thermogravimetric loss) compared to the control is attributed to the nucleating effect of nano-SiO_2_ on the enhanced generation of hydration products [27].

TGA data indicate that the KC activation system displays more significant weight loss of hydration products, signifying the highest activation level. The NHC activation system follows with a slightly lower weight loss, while the KH and NH activation systems exhibit a weight loss of only about 5%.

### 3.4. Microstructure and EDS Energy Spectrum Analysis

**Figure 8:**
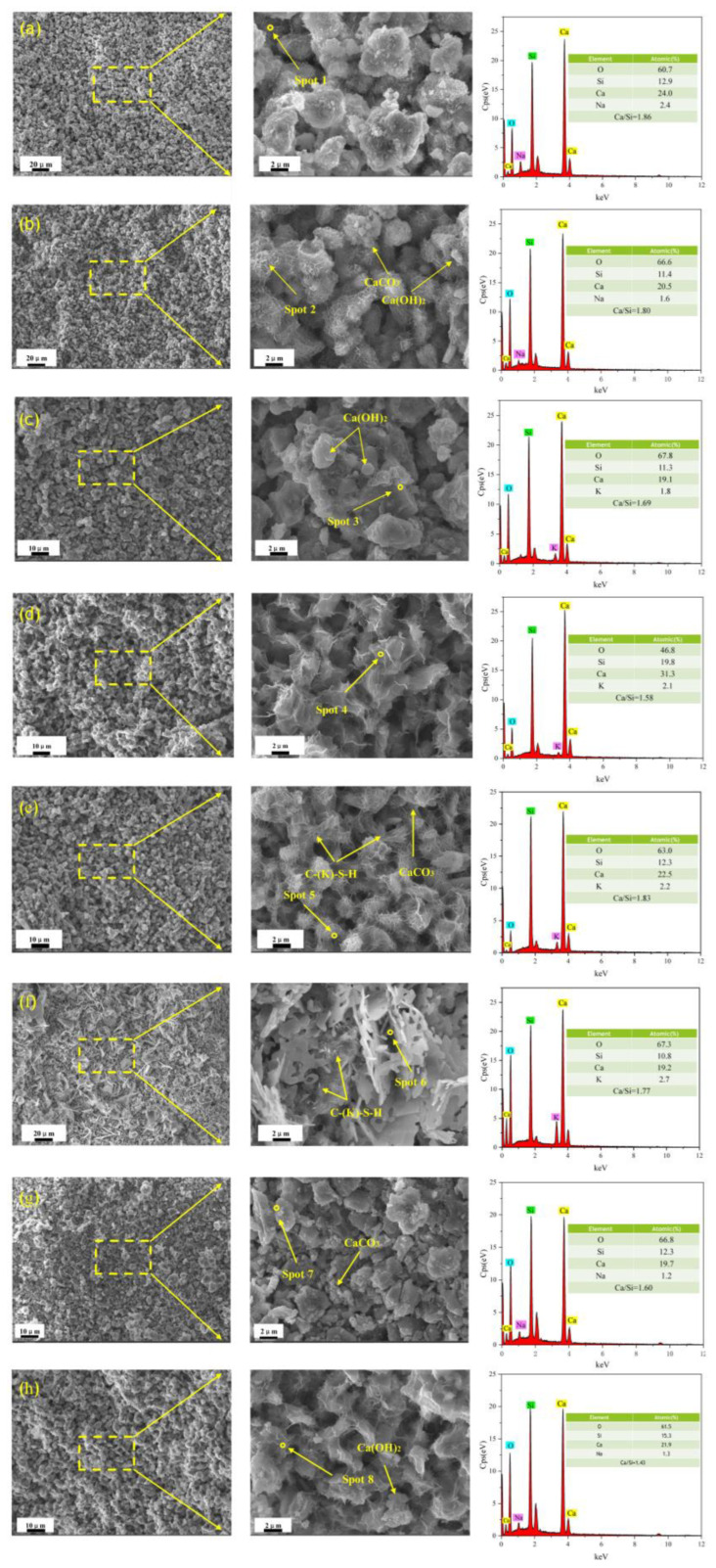
SEM images and EDS analysis of alkali-activated pastes for 28 days. (**a**) NHC, (**b**) NHC+Si, (**c**) NH, (**d**) NH+Si, (**e**) KC, (f) KC+Si, (**g**) KH, and (**h**) KH+Si.

The secondary electron micrographs of the microstructures of γ-C_2_S samples hydrated for 28 days under different activation systems are presented in Figure 8. The middle images is the enlarge images of the yellow box in the pictures on the left. EDS spectroscopic data indicate that many Ca^2+^ ions in C-S-H are replaced by Na^+^ and K^+^ ions to form C-(R)-S-H gels.

The variation in the structures of the C-(R)-S-H gels can be attributed to the type of activator used and the difference in the calcium-to-silicon ratio of the products. As shown in Figure 8a, the network structure of C-(R)-S-H is observed in NHC-activated hydrated pastes, but a number of unhydrated γ-C_2_S particles are still visible. In Figure 8b, the incorporation of nano-SiO_2_ transforms the C-(N)-S-H structure. The surface of the γ-C_2_S particles is covered with numerous network-shaped C-(N)-S-H, which connects to the overall structure. Additionally, hexagonal plate-like Ca(OH)_2_ and granular-shaped CaCO_3_ are observed filling the interstitial spaces. Additionally, the hydration products of the NH-activated paste are primarily composed of granular C-(N)-S-H gels. Despite this, many pores and unhydrated γ-C_2_S particles are discovered in the space, indicating a loose microstructure. With the inclusion of nano-SiO_2_, the C-(N)-S-H gel within the NH+Si-activated paste gradually transforms into a fluffy structure. The hydration products in the KC+Si-activated paste are predominantly lamellar and exhibit a distinct network-like interconnection, suggesting their origination from network-like structures. A large number of unhydrated γ-C_2_S particles are observed in the KH sample, indicating a low degree of hydration. Similarly, the sheet structure of C-(K)-S-H gels is observed in KH+Si-activated paste.

EDS data show an exchange of Na^+^ and K^+^ in the activator with Ca^2+^ ions of the C-S-H gel. The exchanged ions attach to the Si-OH bond, forming Si-ONa or Si-OK groups, disrupting the vitreous network structure, and generating the secondary hydration product C-(R)-S-H [28]. Meanwhile, the incorporation of SiO_2_ nanoparticles promotes the generation of C-(R)-S-H gels with a low Ca/Si ratio, which is attributed to the decalcification of C-(R)-S-H [29]. The high reactivity of SiO_2_ nanoparticles triggers the interaction between Si-OH groups on its surface and Ca(OH)_2_ with the pozzolanic effect. This interaction subsequently diminishes the solubility of Ca(OH)_2_ in the hydrated slurry. Moreover, it curbs the interplay between C-S-H and Ca(OH)_2_ and impedes the transformation of hydrated products into high Ca/Si phases [30].

### 3.5. ^29^Si MAS NMR Analysis

**Figure 9:**
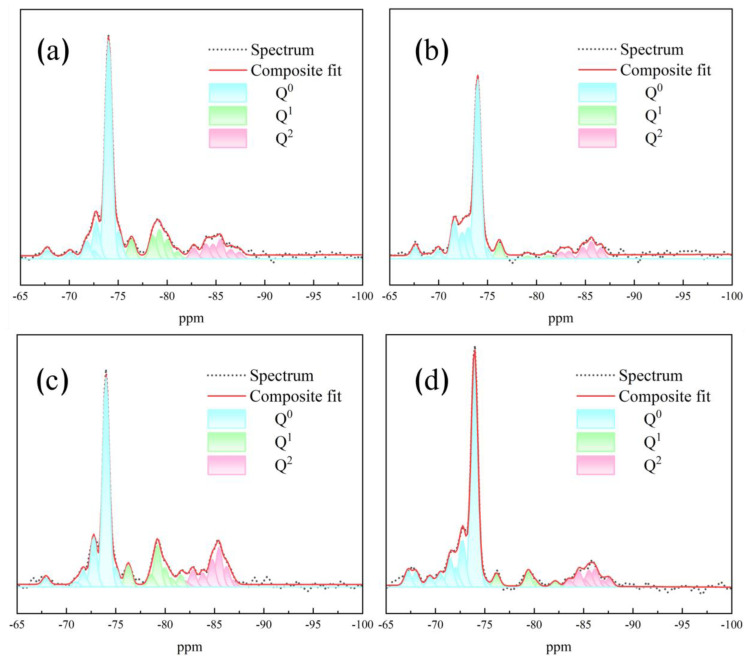
Spectra of ^29^Si MAS NMR and its deconvolution results for each hydration group. (**a**) NHC+Si, (**b**) NH+Si, (**c**) KC+Si, and (**d**) KH+Si.

The ^29^Si MAS/NMR spectra for hydration at 28 days under different activation systems are presented in Figure 9, including experimental data, fitted curves, and deconvolution data. Q^0^ signifies the monomeric orthosilicate anion SO_4_^4−^. The resonances of Q^1^ and Q^2^, respectively, correspond to silicon–oxygen tetrahedra with dimeric straight-chain end groups and intermediate groups, reflecting the end groups and dimers of the silicate chains formed in the C-S-H phase. Q^3^ and Q^4^ represent chain branching sites and fully (three-dimensional) cross-linked groups, respectively. The variation in the strength of the Q^1^ and Q^2^ units relies on the disparity in the degree of silicate polymerization within the C-S-H gels.

The ^29^Si MAS/NMR data of all activated samples indicate the presence of three peaks in each of them. The primary peak appears at −73.9 ppm (Q^0^), corresponding to the chemical shift of unreacted γ-C_2_S [31]. The chemical shifts of Q^1^ and Q^2^ are consistent across all hydrated samples (at −79.0 ppm and −85.2 ppm, respectively), representing characteristic signals for C-S-H gels. The findings demonstrate that the structure of the C-(R)-S-H phase (i.e., the arrangement of silica–oxygen tetrahedra) is similar to that of the C-S-H phase, containing primarily Q^1^ (end groups) and Q^2^ (bridging tetrahedra). The differences in Ca/Si ratios account for the fluctuations in signal intensities of Q^1^ and Q^2^ among different activations. As the Ca/Si ratio increases, the signal intensity of Q^2^ decreases while that of Q^1^ increases.

Deconvolution data were calculated using Peak Fit 4.12 software to determine the quantitative information about C-S-H and unreacted γ-C_2_S in activated samples [32]. The method for calculating the degree of hydration (α) involved subtracting the percentage of unreacted γ-C_2_S (i.e., Q^0^) from 100. The average silica chain length (MCL) was determined using Equation (1) [7]:

(1) $$\mathrm{MCL}=2\times \frac{\left[I\left(Q^{1}\right)+I\left(Q^{2}\right)\right]}{I\left(Q^{1}\right)}$$

Table 1 presents the deconvolution findings for each hydrated paste. The results show that the hydration degree of γ-C_2_S pastes is enhanced by the presence of an alkaline activator and nano-SiO_2_. Specifically, the KC+Si-activated paste achieves a hydration level of 47.78% at 28 days, while the NH+Si-activated paste exhibits the lowest hydration with only 13.02%.

**Figure 10:**
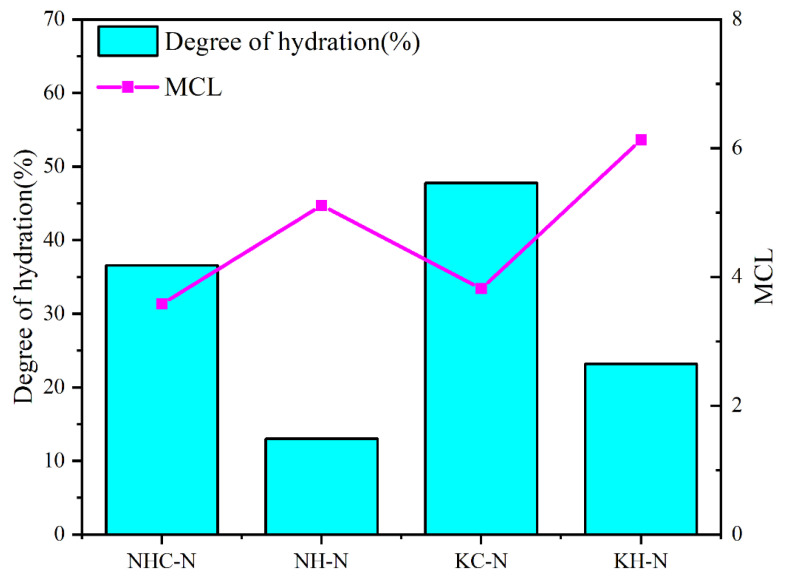
The hydration degree (α) and silica chain length (MCL) of C-(R)-S-H from ^29^Si NMR spectra.

The MCL serves as an effective indicator of the degree of silicate polymerization within the C-(R)-S-H phase. As shown in Figure 10, despite the NH+Si- and KH+Si-activated pastes having lower C-S-H contents, they exhibit higher average silicate chain lengths (5.11 and 6.13, respectively). This phenomenon can be attributed to the low Ca/Si of the NH+Si- and KH+Si-activated pastes, which helps maintain the integrity of the [SiO_4_]^4−^ tetrahedral structure [33]. As a result, they exhibit a heightened level of silicate polymerization. Additionally, the results show a marginally superior polymerization degree of C-(K)-S-H gels compared to that of C-(N)-S-H gels. This trend can be attributed to the high capacity of C-S-H gels to retain potassium ions in contrast to sodium ions. Due to the small ionic radius of potassium ions, potassium cations exhibit a more vital coulombic interaction, allowing them to penetrate the C-S-H gel structure and counterbalance the negative charge within the C-S-H layer. Potassium ions and water molecules occupy the parallel channels between the silica tetrahedra layers, which creates notably strong hydrogen bonds, consequently enhancing the aggregation density of C-(K)-S-H gels [9]. In contrast to potassium salts, sodium cation is retained in the C-S-H structure by displacing calcium ions and compensating the OH^−^ group charge in the structure. Nevertheless, they are only retained at the terminus of the C-S-H gel structure, leading to a lower retention capacity than potassium ions [34,35]. Consequently, a variation in signal intensity arises between Q^1^ and Q^2^, which influences the degree of silicate polymerization.

Combined with EDS data, incorporating nano-SiO_2_ induces the decalcification reaction due to its enhanced reactivity. This reaction produces a lower C/S ratio within the C-(R)-S-H structure. The degree of silicate polymerization in the C-S-H phase is closely related to the C/S ratio [36]. C-(R)-S-H with low Ca/Si ratios exhibits long silicate chain lengths. Longer silica chain lengths indicate an elevated level of silicate polymerization in the C-(R)-S-H phase. Consequently, the incorporation of nano-SiO_2_ can significantly enhance the degree of silicate polymerization of C-(R)-S-H gels, causing the structures to be favorable and thereby contributing to the improved mechanical properties [26,37]. In addition, the formation of C-S-H phases with low C/S ratios decreases the alkali concentration in the pore solution, generating more C-S-H phases to accommodate the lower overall C/S ratio in the system [38].

## 4. Conclusions

This study investigates the combined effect of chemical activators and nano-silica on the hydration behavior of γ-C_2_S. The results prove that the reactivity of γ-C_2_S activated with chemical activators can be significantly enhanced by nano-SiO_2_. The main conclusions of this study are summarized as follows:

1. The highest total heat release during the first 3 days of hydration is observed with the Na_2_CO_3_ activator, followed by the K_2_CO_3_ and KOH activators, and the lowest cumulative heat release is for the NaOH activator. The cumulative exothermic capacity of the chemically activated samples can be significantly increased with the inclusion of nano-SiO_2_.
2. The primary products of γ-C_2_S slurry activated with chemical activators are C-(R)-S-H gels, calcium carbonate, and portlandite. Nano-SiO_2_ primarily serves as a seed to provide nucleation sites for the growth of γ-C_2_S hydration products. It does not alter the hydration product type but reduces the Ca/Si ratio of the hydrated product (C-(R)-S-H gel). The morphology of C-(R)-S-H gels is influenced by the Ca/Si and the type of chemical activator.
3. ^29^Si NMR spectroscopy shows that the C-(R)-S-H phase mainly comprises Q^1^ (end chain) and Q^2^ (bridging silicate tetrahedra) with a structure similar to C-S-H. Variations in the calcium–silica ratio impact the degree of C-(R)-S-H polymerization. The decalcification reaction induced by nano-SiO_2_ can effectively increase the degree of C-(R)-S-H polymerization and form favorable structures.
4. K_2_CO_3_ exhibits the most effective activation effect on γ-C_2_S, while NaOH displays the worst. It is confirmed by measurement methods (TGA, SEM, and NMR) that KC+Si-activated γ-C_2_S has the highest total hydration after 28 days of sample reaction. Under the combination of K_2_CO_3_ and nano-SiO_2_ (KC+Si group), the 28-day hydration degree of γ-C_2_S slurry can reach up to 48%. The NHC+Si group achieves approximately 37% hydration, while the KH+Si and NH+Si groups are only about 20% after the same hydration duration.

In this sense, it is concluded that the non-hydraulic γ-C_2_S can be transformed into reactive minerals by the combined effect of chemical activation and crystal species activation. This will provide a low-pollution and low-risk way to enhance the activity of environmentally friendly cementitious materials (such as cementitious materials primarily composed of low-lime calcium silicates and steel slag-based cementitious materials) and a theoretical basis for the preparation of belite-based low-carbon gelling materials.

## Figures and Tables

**Table 1:** Deconvolution results of ^29^Si MAS/NMR spectra for γ-C_2_S slurry hydration for 28 days.

Sample Name	IQ^n^(%)			MCL	α (%)
	IQ^0^	IQ^1^	IQ^2^		
NHC+Si	63.42	20.43	16.15	3.58	36.58
NH+Si	86.98	5.10	7.92	5.11	13.02
KC+Si	52.22	25.00	22.78	3.82	47.78
KH+Si	76.82	7.56	15.62	6.13	23.18

## Data Availability

Data sharing is not applicable.
